# Evaluation of interleukin 8 and interleukin 10 cytokines in liquid based cervical cytology samples

**DOI:** 10.11604/pamj.2019.32.148.16314

**Published:** 2019-03-26

**Authors:** Daniel David Osiagwu, Alfred Ederialo Azenabor, Abimbola Akinduro Osijirin, Philomina Ikuoyah Awopetu, Folasade Ruth Oyegbami

**Affiliations:** 1 Department of Medical Laboratory Science, University of Lagos, Nigeria; 2 Lagos University Teaching Hospital, Lagos, Nigeria

**Keywords:** Cytokines, cervical cancer, inflammation

## Abstract

**Introduction:**

inflammatory cytokines have been associated with various cancers, including cervical cancers. Interpreting cytokine expression in liquid based cervical samples is quite challenging. This study is aimed at evaluating the levels of interleukin 8 and 10 in liquid based cervical samples.

**Methods:**

this is a descriptive analytical study carried out on eighty five (85) subjects aged between 23 and 68 years. Cervical samples were collected in liquid based medium and smears later examined after staining with Papanicolaou technique. These were categorized into low grade intra-epithelial lesion/malignancy, high grade intraepithelial lesion/malignancy according to the degree of dyskaryosis. Concentrations of interleukin 8 and interleukin 10 in the samples were determined by enzyme linked immunosorbent assay.

**Results:**

the mean age, standard deviation (SD) of the study subjects were 40.6 (7.8) years. A total number of 79 females (92.9%) were negative for intra-epithelial lesion/malignancy (NILM), while 4 (4.71%) and 2 (2.35%) were positive for low grade intra-epithelial lesion/malignancy (LILM) and high grade intra-epithelial lesion (HILM) respectively. While mean levels of interleukin 8 increased with the degree of malignancy, (107.27 ± 11.88pg/ml) in LILM, (114.80 ± 2.12pg/ml) in HILM when compared with NILM (88.39 ± 18.06pg/ml), (f = 0.700, p = 0.018); the mean levels of interleukin 10 was comparable between these groups (p ≥ 0.05). Pearson correlation coefficient analysis showed a negative association between interleukin 8 and interleukin 10 (r = -1.999, p = 0.000) in LILM.

**Conclusion:**

interleukin 8 cytokines in cervical cancer is associated with the degree of malignancy. Possible anti-inflammatory effect of interleukin 10 was not observed.

## Introduction

Cervical cancer is a huge challenge to the health systems of several countries in the world [1], affecting the quality of life and loss of work hours of millions of women in the age group of 40 to 65 years of age while more than 50% of them die because of late diagnosis in spite of expensive treatment [2, 3]. Cervical cancers develop when normal cells in the cervical epithelium undergo abnormal changes and spread deeper into cervix [4]. These mostly arise within the transformation zone at the squamo-columnar junction of the cervix [5, 6]. According to the World Health Organization in 2017, cervical cancer was reported to be the second most common cancer among women worldwide [7]. In Nigeria, an estimated 10,000 new cases of cervical cancer and 8000 deaths due to the disease are recorded among women yearly [8]. It is estimated that over 99% of cervical cancer cases are caused by human papilloma virus [9] and this organism show strong association with Herpes Simplex Virus Type 2(HSV-2) [10] Sexually transmitted infections of the cervix, such as HSV-2 could result into a complex interplay of both the systemic and local immune responses. The cervix is unique in that it naturally produces cervical secretions by columnar cells and these secretions appear to be rich in immunoregulatory proteins including cytokines. At the local level and with the complexity of the cervical environment, it is likely that numerous cytokines are locally expressed and essential for the proper control of pathogens. Invariably, it has also been observed that circulating cytokine levels in local tissues is altered in a number of cancers, including gynecological [11].

It is well established that cancer can be promoted and/or exacerbated by inflammation and infections. Chronic inflammation provides a tumor supporting environment that participates in the neoplastic process and the mechanisms that link infection, immunity, inflammation and cancer include cytokines produced by activated innate immune cells that stimulate tumor growth and progression [12]. In addition, soluble mediators produced by cancer cells activate inflammatory cells, which further stimulate tumor progression. Whilst previous study by Wu *et al.* has shown that in carcinogenesis, excess interleukin8 (IL-8) indicates growth and or metastasis of the tumour [13], the anti-inflammatory side of the equation is less well known. It is pertinent to note that anti- inflammatory cells also produce cytokines that can limit tumor growth [14]. This includes interleukin 10, which down regulate immune responses, providing natural immune suppression with therapeutic potential. It is instructive to note that cervical concentrations of cytokines have been shown not to correlate with plasma concentrations [15], thereby fuelling an intense debate on its diagnostic usefulness. Until now, there have been no reported studies that evaluated the pattern of occurrence of cytokines in cervical samples in sub Saharan Africa. This study was aimed at evaluating the levels of pro-inflammatory and anti-inflammatory cytokine milieu in liquid based cervical samples. Another objective was to explore a possible association of these cytokines with the degree of dyskaryosis.

## Methods

This is a descriptive analytical study carried out at the Lagos University Teaching Hospital, Lagos, Nigeria. Consenting patients were recruited consecutively over a six month period. One hundred (100) females attending the Dept. of Anatomic and Molecular Pathology, of Lagos and the University Teaching Hospital, Idi-Araba, Lagos were initially recruited, out of which eight-five (85) of them fully participated in the study. They were between the ages of 23-68 years of age. Ethical approval was obtained from the Research and Ethics Committee of the Hospitals and informed consent was obtained from the participants or their proxies prior to the study. A well-structured questionnaire was used to obtain information pertaining to their demographic bio data, drug history and menstrual cycle. Inclusion criteria: are women between the ages of 20 and 70 years as well as those not previously being managed for cervical cancers Exclusion criteria: include those currently on anti-inflammatory drugs or immune modulators, pregnant and menstruating females, those positive and currently being treated for endocervical infections such as Clamydia trachomatis, Neisseria gonorrhea, HPV etc.

**Sample collection and processing:** sample collection from the participants involve the individuals laying in a supine position in a gynaecological examination chair. Firstly, Rovers cervix brush was used (Liqui PrepTM.Rovers Inc, USA) to collect the sample, after collection, the head of the brush is detached and placed into the liquid preparative collection vial (Liqui PrepTM LGM, Inc, USA) containing a liquid base medium. The sample in the vial was then thoroughly mixed in the liquid base medium, poured into a centrifuge tube with an equal volume of cleaning solution((Liqui PrepTM LGM, Inc, USA). The fluid was collected and centrifuged for 10 minutes at 1400 rpm; the supernatant was removed and stored in aliquots at -80°C until assayed. Smears were made from the residue after mixing with cellular base solution ((Liqui PrepTM LGM, Inc, USA) and stained by Papanicolaou staining technique and reported.

**Biochemical analysis:** the cytokines (interleukin 8 and 10) were analyzed using standard Elisa method, employing WKEA kit (China) and the concentrations of which were extrapolated from absorbance of the standard curves generated.

**Statistical analysis:** the data generated were analyzed using SPSS version 17. independent student's test was used to compare groups. Within group analysis and between group analyses was done using one way analysis of variance (ANOVA). Pearson's correlation coefficient determination was used to evaluate the degree of association. Quantitative data were expressed as mean ± SD. Probability values of less than 0.05 were considered to be statistically significant.

## Results

**Figure 1:**
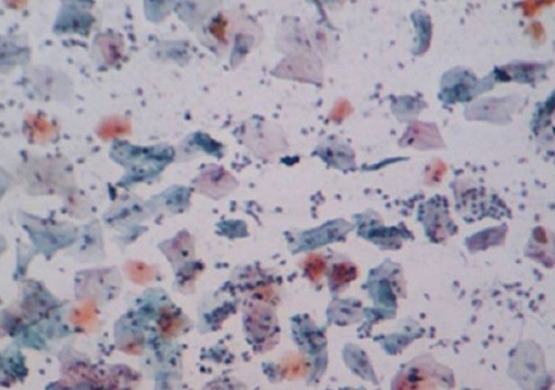
negative intraepithelial lesion/malignancy on an inflammatory background

**Figure 2:**
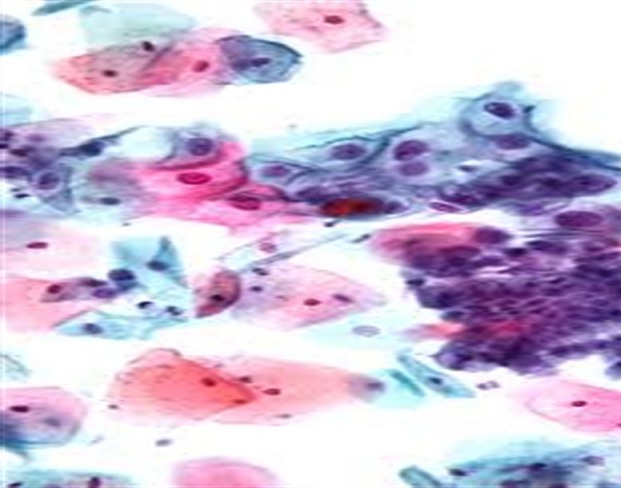
low grade intraepithelial lesion/malignancy on atrophic smear

**Figure 3:**
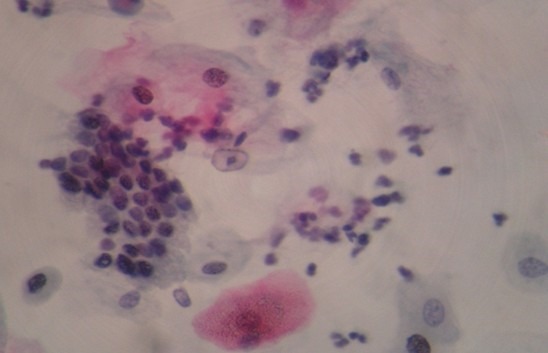
high grade intraepithelial lesion/malignancy on atrophic smear

The mean age, standard deviation (SD) and age range of the study subjects were 40.6 (78 ) years and 23-68 years respectively. A total number of 79 females (92.9%) were negative for intra- epithelial lesion/malignancy (NILM), while 4 (4.71%) and 2 (2.35%) were positive for low grade intra-epithelial lesion/malignancy (LILM) and high grade intra-epithelial lesion (HILM) respectively. While mean levels of interleukin 8 increased with the degree of inflammation, (107.27 ± 11.88pg/ml) in LILM, (114.80 ± 2.12pg/ml) in HILM when compared with NILM (88.39 ± 18.06pg/ml), (f = 0.700, p = 0.018) the mean levels of interleukin 10 was comparable between these groups (Table 1). The expression of the cytokines in cervical samples showed no association with age all the three groups (Table 2). Association of cytokines (interleukin 8 and interleukin 10) in different degree of dyskaryosis shows an inverse association in low grade intra-epithelial lesion/malignancy (LILM) (Table 3). The slide picture of a negative intraepithelial lesion/malignancy on an inflammatory background is shown in Figure 1. Low grade intraepithelial lesion/malignancy on atrophic smear (Figure 2). High grade intraepithelial lesion/malignancy on atrophic smear (Figure 3).

**Table 1:** levels of Interleukin 8 and Interleukin 10 in Negative, low grade and high grade intra- epithelial lesion/malignancy

Cytokines (pg/ml)	NILM n = 79	LSIL	HSIL		
		n = 4	n = 2	f values	p-values
IL – 8	88.39 ± 18.06	107.27 ± 11.88	114.80±2.12	0.700	0.018**
IL – 10	82.14 ± 59.85	99.11 ± 69.01	57.19±11.99	0.696	NS

Values are presented as Mean ± SD. p values calculated using one way analysis of variance.

** Significant; NS, Not significant. IL-8 (Interleukin 8), IL-10 (Interleukin 10), NILM (Negative intra -epithelial lesion/malignancy), LSIL (Low grade intra-epithelial lesion/malignancy), HSIL (high grade intra-epithelial lesion/malignancy)

**Table 2:** association of age with the cytokines in different degree of dyskaryosis

Degree of dyskaryosis	Interleukin- 8 r (p)	Interleukin – 10 r (p)
NSIL (n = 79)	-0.045 (0.693)	0.090 (0.432)
LSIL (n = 4)	-0.144 (0.856)	0.180 (0.820)
HSIL (n = 2)	-1.000 (N/A)	1.000 (N/A)

P values are calculated using Pearson Correlation Coefficient. N/A-Not Applicable

**Table 3:** association of cytokines in different degree of dyskaryosis

Degree of Dyskaryosis	IL - 8 Vs. IL – 10 (r)	p values
NSIL	-0.888	0.443
LSIL	-0.999	0.001*
HSIL	-1.000	N/A

P values are calculated using one way analysis of variance.

* Significant

## Discussion

The levels of cytokines in liquid based cervical samples have not been objectively assessed in sub Saharan Africa. This study attempted to explore the levels of proinflammatory and anti-inflammatory cytokine milieu in liquid based cervical samples. This was in bid to establish a possible association with the degree of dyskaryosis in cervical cancer. This study has shown that the presence of pro-inflammatory cytokine (Interleukin 8) and anti-inflammatory cytokine (interleukin 10) are easily detected in cervical samples in liquid based medium. This observation is in tandem with a previous report by Vidal *et al*. Although liquid based medium remove unwanted materials such as inflammatory exudates, blood and mucus from cells when mixed with cleaning solution [16]; their expression in cervical samples were not hindered as shown in this study. It is also interesting to note that interleukin-8 cytokine was related to the severity of cervical neoplasia, when the cytokine levels in subjects with high grade intra-epithelial neoplasia were compared with low grade neoplasia and subjects without neoplasia. Our findings from this study agree with a previous study reported elsewhere [17]. It is important to mention that the cervical milieu could also be influenced by environment factors such as infectious agents as well the carcinogens. However, this could not be ascertained in this study as the presence of endocervical infections were ruled not out prior to analysis. Interleukin-8, initially shown to be a chemoattractant for lymphocytes and neutrophil has been found to act multifunctionally by inducing neutrophil infiltration to the size of infection and act as a mediator of inflammation at the site of infection [18]. The collateral damage caused by this type of inflammation accumulates slowly and sometimes asymptomatically and leads to further deterioration. The inflammatory system can be linked to a sprinkler system that prevents fire from spreading in a building, while the intention is to limit damage and restore function (positive), the response itself can cause significant harm. One undesirable consequence of inflammation is that some enzymes and toxic products contained in phagocytic tissues are inevitably released, damaging cells and tissues. Interestingly, the mean levels of interleukin 10 in cervical samples did not differ between subjects negative for malignancy and those with both low and high grade intraepithelial lesion, although insignificantly higher levels were noticed in the low grade group. The potential anti-inflammatory role of interleukin 10 is evidenced in the group with low grade lesion, as shown in our correlation studies with interleukin 8. Interleukin 10 is also called cytokine synthesis inhibitory factor because it inhibits the production of other inflammatory cytokines as well as macrophages. Increased concentrations of interleukin 10 in LILM could possibly be due to a compensatory mechanism for the inflammatory process induced by interleukin 8, however, this seems not to be enough to suppress a higher degree of inflammation. Barring the limitations of positive cases, the inverse pattern of pro-inflammatory cytokine and anti-inflammatory cytokine expected could not be ascertained in the high grade group studied. We have also shown that age is not a determinant in the expression of cytokines in the cervical samples.

**Limitation of study:** this study was limited by the few number of positive cases, but has provided a thrust for a larger and more prospective studies.

## Conclusion

Interleukin 8 and Interleukin 10 cytokines are expressed in the cervical samples. The level of interleukin 8 is associated with the degree of cervical cancer neoplasia.

### What is known about this topic

- Inflammatory cytokines have been associated with various cancers, including cervical cancers and these were hitherto assessed in peripheral blood samples.

### What this study adds

- This study has been able to show the expression of cytokines in liquid based cervical cytology samples; the expression of which reflects the degree of dyskaryosis.

## Competing interests

The authors declare no competing interests.
